# Potential linkage of different phenotypic forms of childhood strabismus to a recessive susceptibility locus (16p13.12-p12.3)

**Published:** 2011-04-19

**Authors:** Arif O. Khan, Jameela Shinwari, Nada Abu Dhaim, Dania Khalil, Latifa Al Sharif, Nada Al Tassan

**Affiliations:** 1 Division of Pediatric Ophthalmology, King Khaled Eye Specialist Hospital, Riyadh, Saudi Arabia; 2 Department of Genetics, King Faisal Specialist Hospital and Research Center, Riyadh, Saudi Arabia

## Abstract

**Purpose:**

To perform linkage analysis on an inbred family with members who exhibit different phenotypic forms of childhood strabismus.

**Methods:**

Prospective clinical examination and linkage analysis.

**Results:**

Three of the ten siblings and their cousin each had a different phenotypic form of childhood strabismus: infantile esotropia with convergence excess, esotropia associated with anisometropic amblyopia, unilateral esotropic Duane syndrome, and monocular elevation deficiency. Linkage analysis for the four strabismic individuals, an unaffected sibling, and the unaffected parents identified a single disease locus on chromosome 16p13.12-p12.3 (Ensembl cytogenetic band) with a 2.5 maximum logarithm of odds score. The region is 6 MB in size and comprises 80 genes.

**Discussion:**

Linkage analysis in this unique family suggests that childhood strabismus can be recessive and that different phenotypic forms of childhood strabismus can share the same underlying genotype.

## Introduction

Strabismus (ocular misalignment) is usually comitant (the same in different gaze positions) rather than incomitant (varying with different gaze positions) and affects up to 4% of the general population [1-3]. Most isolated strabismus is sporadic, but familial cases have been described for virtually all types and some types are more commonly familial than others [2,3]. Although all forms of early childhood strabismus have in common a disruption of normal binocular vision, there are distinct phenotypes. Comitant esotropia (inward deviation) is most common form in most populations [1-4] and includes infantile esotropia, convergence excess esotropia, and sensory esotropia. Infantile esotropia is a large-angle esotropia not typically associated with significant refractive error. Convergence excess esotropia refers to a significantly larger esotropia during near fixation that is not related to uncorrected hyperopic refractive error (unlike refractive accommodative esotropia, which is often familial and is the most common form of childhood esotropia) [3]. Sensory esotropia is from poor vision in one eye and often is related to significant uncorrected refractive error in the non-preferred eye. Congenital incomitant strabismus is less common than comitant strabismus and includes phenotypes such as monocular elevation deficiency and Duane retraction syndrome [4]. In monocular elevation deficiency, the affected eye is hypotropic (lower than the fellow eye) and unable to elevate beyond the midline, although elevation can sometimes be elicited via an ocular reflex that occurs during forced lid closure (Bell phenomenon). In Duane retraction syndrome, the most common form of congenital incomitant strabismus [4], the lateral rectus muscle of the affected eye has subnormal innervation from the sixth cranial nerve and variable inappropriate innervation from the ipsilateral third cranial nerve.

Despite the frequency of strabismus in the general population, the genetics of common forms of comitant and incomitant strabismus are not well described [1-3]. One confounding variable in previous genetic studies of strabismus is potential heterogeneity of cause when unrelated patients are studied [1-3]. Different genotypes can underlie the same strabismus phenotype and environmental factors such as prematurity can also play a strong independent role. The identification and study of multiple cases from a single family is a strategy that could minimize this issue. Also confounding previous studies of the genetics of common strabismus is the fact that studies sometimes group different strabismus phenotypes together (e.g., infantile esotropia and refractive accommodative esotropia) when in fact they may be distinct conditions rather than phenotypic variability for the same underlying cause [5-7]. Again, the identification and study of multiple cases from a single family is a strategy that would minimize this issue as in a single family it is more likely for different strabismus phenotypes to have the same underlying etiology.

In this study, we describe a single unique inbred family that has multiple members affected by different forms of childhood strabismus and perform linkage analysis to explore the possibility that a single recessive susceptibility locus underlies their phenotypes.

## Methods

This study was approved by our institutional review boards and adhered to research adhered to the tenets of the Declaration of Helsinki. Full informed consent was obtained from the family after explanation of the nature of the study.

### Clinical

A consanguineous family with four members affected by childhood strabismus was identified from the pediatric ophthalmology practice of one of the authors (A.O.K.) and was invited to participate in the study. Each available family member underwent complete ophthalmic examination with attention to ocular motility both before and after pharmacologic cycloplegia (cyclopentolate 1%) by an ophthalmologist with strabismus experience (A.O.K.).

### Genetic

Blood samples were obtained with informed consent from family members. DNA was extracted from 3 ml of whole blood using Gentra Systems (QIAGEN, Valencia, CA) according to the manufacturer’s conditions. The 10K single nucleotide polymorphism (SNP) genotyping was performed as detailed by Affymetrix (Santa Clara, CA) on the GeneChip® Human Mapping 10K Array Xba 142 2.0. The SNP genotypes were called using Affymetrix GCOS 1.4 software with an overall SNP call rate of 95%–99%. Multipoint logarithmic odds (LOD) score calculations were performed with the Allegro module of the EasyLinkage software package [8] assuming an autosomal recessive mode of inheritance with 100% penetrance and disease allele frequency of 0.01%.

## Results

### Clinical

**Figure 1:**
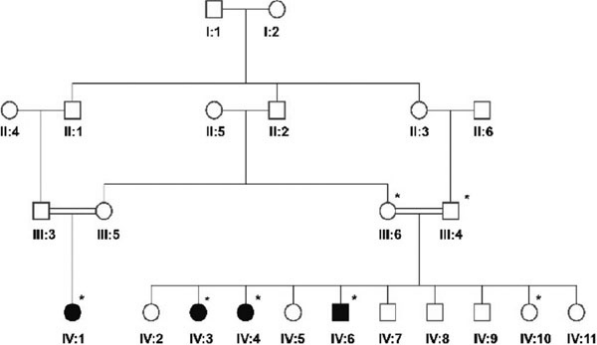
Pedigree. Four individuals from this consanguineous family had early childhood strabismus. Individuals included in the linkage analysis are indicated by asterisks.

The family pedigree in shown in Figure 1 (starred individuals participated in the study). The asymptomatic parents (III:6, III:4) and one asymptomatic sibling (IV:10) were examined and confirmed to have no significant ophthalmic findings. The four individuals with childhood strabismus (IV:1, IV:3, IV:4, and IV:6) are summarized below:

#### IV:1

**Figure 2:**
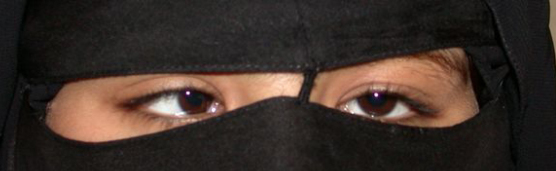
Patient IV:1 – infantile esotropia with convergence excess. The patient is shown fixating with her left eye. The right eye straightens and the left eye turns inward when she fixates with her right eye (not shown).

This 18-year-old girl (Figure 2) had inward ocular deviation noted within the first few months of life and an unremarkable birth history. She had no treatment before presentation. Best corrected visual acuity was 20/50 in her right eye (OD) and 20/20 in her left eye (OS). Ophthalmic examination was significant for 45 prism diopters (PD) esotropia at distance and 75 PD esotropia at near without duction limitations. Cycloplegic refraction and fundus examination were unremarkable. Her history and examination were consistent with infantile esotropia combined with convergence excess and amblyopia OD.

#### IV:3

This 19-year-old girl had inward ocular deviation noted within the first few years of life and an unremarkable birth history. She had undergone strabismus surgery OS for her eye turn a few years before presentation. Best-corrected visual acuity was 20/20 OD and 20/200 OS. Ophthalmic examination was significant for 8 PD esotropia at distance and 15 PD esotropia at near without duction limitations. Cycloplegic refraction revealed +1.50 diopters OD and +5.00 diopters OS. Fundus examination was unremarkable. Her history and examination were consistent with childhood sensory esotropia secondary to anisohyperopic amblyopia OS.

#### IV:4

**Figure 3:**
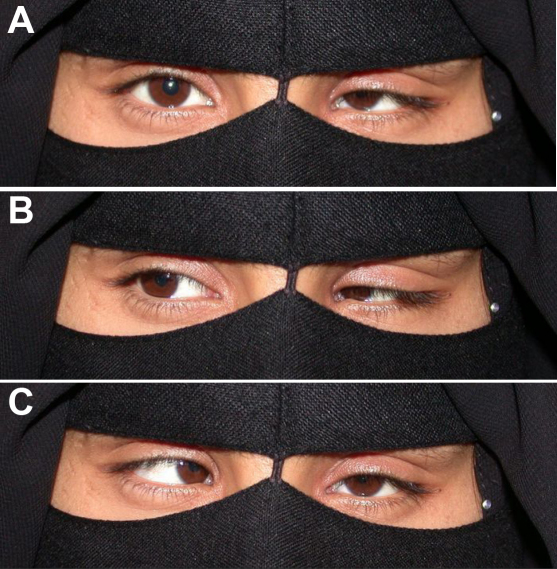
Patient IV:4 – left esotropic Duane retraction syndrome. **A**: In primary position, a left esotropia can be appreciated. There is also slight ptosis. **B**: During attempted right gaze, the left eye retracts. **C**: During attempted left gaze, there is a significant abduction defect of the left eye.

This 18-year-old girl (Figure 3) had inward ocular deviation noted within the first few years of life and an unremarkable birth history. She had no treatment before presentation. Best-corrected visual acuity was 20/20 OD and 20/400 OS with eccentric fixation OS. Ophthalmic examination was significant for 45 PD esotropia at distance and near with abduction limitation OS (−3, with −4 being no duction), globe retraction during adduction OS, and true mild ptosis OS. Cycloplegic refraction and fundus examination were unremarkable. Her history and examination were consistent with congenital Duane retraction syndrome OS with mild true ptosis and amblyopia OS.

#### IV:6

**Figure 4:**
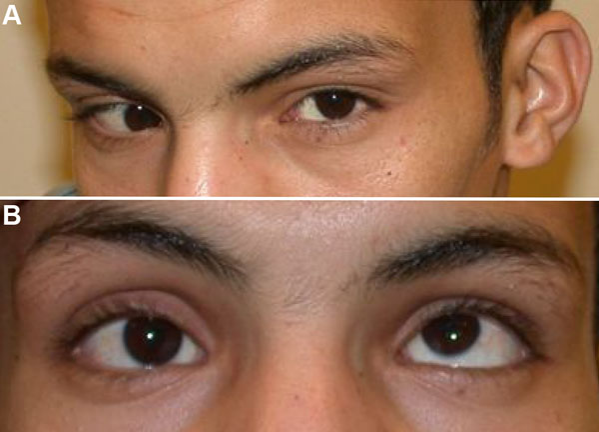
Patient IV:6 – right monocular elevation deficiency. **A**: The patient prefers a slight chin down head position with a slight right face turn. **B**: The elevation deficiency of the right eye is obvious during attempted upgaze.

This 17-year-old boy (Figure 4) had misaligned eyes noted in the first few years of life and an unremarkable birth history. He had no treatment before presentation. He preferred a moderate chin-down head position with a slight right head tilt. Best-corrected visual acuity was 20/30 OD and 20/20 OS. Ophthalmic examination was significant for 25 PD esotropia and 45 PD right hypotropia at distance and near with −2 supraduction limitation OD. There was a good Bell phenomenon to forced lid closure in both eyes. Cycloplegic refraction and fundus examination were unremarkable with no evidence for torsion in either eye. His history and examination were consistent with congenital monocular elevation deficiency OD.

### Genetic

**Figure 5:**
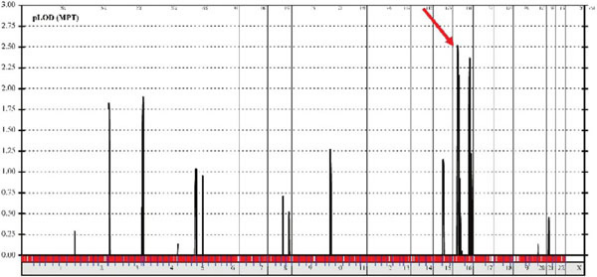
Linkage analysis. Linkage analysis assuming an autosomal recessive mode of inheritance revealed a single maximum peak on chromosomes 16 with a 2.5 logarithm of the odds score (arrow).

Multipoint linkage analysis of family (both unaffected parents [III:6 and III:4 ], all four strabismic individuals [IV:1, IV:3, IV:4, and IV:6], and one unaffected sibling [IV:10]) identified a single disease locus on chromosome 16p13.12-p12.3 (Ensembl project genome database cytogenetic band) with a maximum logarithm of odds (LOD) score of 2.5 (Figure 5). The region is 6 MB in size and comprises 80 genes.

## Discussion

Careful ophthalmic phenotyping and linkage analysis in this unique inbred family with hereditary strabismus revealed four different forms of childhood strabismus – infantile esotropia combined with convergence excess, sensory esotropia from anisometropia, monocular elevation deficiency, and esotropic Duane retraction syndrome. Linkage analysis suggested that the four strabismic phenotypes linked to a single recessive 6 MB band on chromosome 16p13.12-p12.3. These results suggest that childhood strabismus can be recessive and that distinct childhood strabismus phenotypes can be related to the same underlying genetic locus.

Most advances in understanding the genetics of ocular motility have not been for common forms of comitant or incomitant strabismus but rather have been for rarer forms of congenital incomitant strabismus known as congenital cranial dysinnervation disorders [1,9]. For the recessive forms of these disorders, genetic studies of affected consanguineous families have allowed successful identification of novel genes involved in ocular motility such as paired-like homeobox 2a (*PHOX2A*; recessive congenital fibrosis of the extraocular muscles) [10] and roundabout homolog 3 (*ROBO3*; recessive horizontal gaze palsy with progressive scoliosis) [11]. Consanguineous families more commonly have recessive cause for familial ocular disease and also are not likely to be confounded by non-allelic genetic heterogeneity among affected family members [12]. Thus genetic analysis of such families offers a unique opportunity to uncover recessive loci associated with a familial ocular phenotype, as was the case for the family described in the current study. In addition to being amenable for genetic study, the inbred family that we describe was unique in that multiple family members each had different phenotypic forms of common childhood strabismus, evidence that they can represent phenotypic variability for the same disorder.

Prior genetic studies of common forms of childhood strabismus are limited. Both recessive and dominant linkage of childhood esotropia to 7p22.1 have been reported in one family each, but without differentiation of accommodative esotropia from infantile esotropia [5,6]. Additional susceptibility loci for comitant strabismus such as 4q28.3 and 7q31.2 have been reported, but again various subtypes of strabismus were grouped as a single phenotype among unrelated families who often did not have a large number of affected family members and typically were not consanguineous [7]. Infantile esotropia may best fit a codominant inheritance model [13]. Regarding monocular elevation deficiency, to the best of our knowledge no genetic studies have been published to date. Regarding Duane retraction syndrome, which can be considered a congenital cranial dysinnervation disorder [9], a dominant familial form (DURS2) was linked in a large pedigree to 2q31 [14] and is now known to be caused by heterozygous mutation in chimerin 1 (*CHN1*) [15]. However, heterozgyous *CHN1* mutation is a rare cause of Duane syndrome and is not relevant for most of Duane syndrome patients encountered in pediatric ophthalmology clinical practice [15]. There have been other genes such as sal-like 4 (*SALL4*) [16,17] and homeobox a1 (*HOXA1*) [18] that when mutated cause Duane retraction syndrome with systemic manifestation but again these genes are not associated with the common isolated form of Duane retraction syndrome [19,20]. One previous analysis of pedigrees with familial strabismus suggested that isolated Duane retraction syndrome may in some families be allelic to infantile esotropia [21], a hypothesis that is consistent the findings of our study.

In summary, we suggest that childhood strabismus can be related to a recessive susceptibility locus (16p13.12-p12.3). In addition, our results suggest that distinct childhood strabismus phenotypes (infantile esotropia combined with convergence excess, sensory esotropia from anisometropia, monocular elevation deficiency, and esotropic Duane retraction syndrome) can share a similar underlying genotype.

## References

[1] Engle EC (2007). Genetic basis of congenital strabismus.. Arch Ophthalmol.

[2] Lorenz B (2002). Genetics of isolated and syndromic strabismus: facts and perspectives.. Strabismus.

[3] Paul TO, Hardage LK (1994). The heritability of strabismus.. Ophthalmic Genet.

[4] Curtis TH, McClatchey M, Wheeler DT (2010). Epidemiology of surgical strabismus in Saudi Arabia.. Ophthalmic Epidemiol.

[5] Parikh V, Shugart YY, Doheny KF, Zhang J, Li L, Williams J, Hayden D, Craig B, Capo H, Chamblee D, Chen C, Collins M, Dankner S, Fiergang D, Guyton D, Hunter D, Hutcheon M, Keys M, Morrison N, Munoz M, Parks M, Plotsky D, Protzko E, Repka MX, Sarubbi M, Schnall B, Siatkowski RM, Traboulsi E, Waeltermann J, Nathans J (2003). A strabismus susceptibility locus on chromosome 7p.. Proc Natl Acad Sci USA.

[6] Rice A, Nsengimana J, Simmons IG, Toomes C, Hoole J, Willoughby CE, Cassidy F, Williams GA, George ND, Sheridan E, Young TL, Hunter TI, Barrett BT, Elliott DB, Bishop DT, Inglehearn CF (2009). Replication of the recessive STBMS1 locus but with dominant inheritance.. Invest Ophthalmol Vis Sci.

[7] Shaaban S, Matsuo T, Fujiwara H, Itoshima E, Furuse T, Hasebe S, Zhang Q, Ott J, Ohtsuki H (2009). Chromosomes 4q28.3 and 7q31.2 as new susceptibility loci for comitant strabismus.. Invest Ophthalmol Vis Sci.

[8] Lindner TH, Hoffmann K (2005). easyLINKAGE: a PERL script for easy and automated two-/multi-point linkage analyses.. Bioinformatics.

[9] Gutowski NJ, Bosley TM, Engle EC (2003). 110th ENMC International Workshop: the congenital cranial dysinnervation disorders (CCDDs). Naarden, The Netherlands, 25–27 October, 2002.. Neuromuscul Disord.

[10] Nakano M, Yamada K, Fain J, Sener EC, Selleck CJ, Awad AH, Zwaan J, Mullaney PB, Bosley TM, Engle EC (2001). Homozygous mutations in ARIX(PHOX2A) result in congenital fibrosis of the extraocular muscles type 2.. Nat Genet.

[11] Jen JC, Chan WM, Bosley TM, Wan J, Carr JR, Rüb U, Shattuck D, Salamon G, Kudo LC, Ou J, Lin DD, Salih MA, Kansu T, Al Dhalaan H, Al Zayed Z, MacDonald DB, Stigsby B, Plaitakis A, Dretakis EK, Gottlob I, Pieh C, Traboulsi EI, Wang Q, Wang L, Andrews C, Yamada K, Demer JL, Karim S, Alger JR, Geschwind DH, Deller T, Sicotte NL, Nelson SF, Baloh RW, Engle EC (2004). Mutations in a human ROBO gene disrupt hindbrain axon pathway crossing and morphogenesis.. Science.

[12] Alkuraya FS (2010). Homozygosity mapping: one more tool in the clinical geneticist's toolbox.. Genet Med.

[13] Maumenee IH, Alston A, Mets MB, Flynn JT, Mitchell TN, Beaty TH (1986). Inheritance of congenital esotropia.. Trans Am Ophthalmol Soc.

[14] Appukuttan B, Gillanders E, Juo SH, Freas-Lutz D, Ott S, Sood R, Van Auken A, Bailey-Wilson J, Wang X, Patel RJ, Robbins CM, Chung M, Annett G, Weinberg K, Borchert MS, Trent JM, Brownstein MJ, Stout JT (1999). Localization of a gene for Duane retraction syndrome to chromosome 2q31.. Am J Hum Genet.

[15] Miyake N, Andrews C, Fan W, He W, Chan WM, Engle EC (2010). CHN1 mutations are not a common cause of sporadic Duane's retraction syndrome.. Am J Med Genet A.

[16] Al-Baradie R, Yamada K, St Hilaire C, Chan WM, Andrews C, McIntosh N, Nakano M, Martonyi EJ, Raymond WR, Okumura S, Okihiro MM, Engle EC (2002). Duane radial ray syndrome (Okihiro syndrome) maps to 20q13 and results from mutations in SALL4, a new member of the SAL family.. Am J Hum Genet.

[17] Kohlhase J, Heinrich M, Schubert L, Liebers M, Kispert A, Laccone F, Turnpenny P, Winter RM, Reardon W (2002). Okihiro syndrome is caused by SALL4 mutations.. Hum Mol Genet.

[18] Tischfield MA, Bosley TM, Salih MA, Alorainy IA, Sener EC, Nester MJ, Oystreck DT, Chan WM, Andrews C, Erickson RP, Engle EC (2005). Homozygous HOXA1 mutations disrupt human brainstem, inner ear, cardiovascular and cognitive development.. Nat Genet.

[19] Tischfield MA, Chan WM, Grunert JF, Andrews C, Engle EC (2006). HOXA1 mutations are not a common cause of Duane anomaly.. Am J Med Genet A.

[20] Wabbels BK, Lorenz B, Kohlhase J (2004). No evidence of SALL4-mutations in isolated sporadic duane retraction “syndrome” (DURS).. Am J Med Genet A.

[21] Connell BJ, Wilkinson RM, Barbour JM, Scotter LW, Poulsen JL, Wirth MG, Essex RW, Savarirayan R, Mackey DA (2004). Are Duane syndrome and infantile esotropia allelic?. Ophthalmic Genet.

